# Complete genome sequences of *Rhodococcus equi* phages CoffeeBean, Dorin, Francesca, Madraxi, and Tonitrus

**DOI:** 10.17912/micropub.biology.001491

**Published:** 2025-03-16

**Authors:** Orin R. Daspit, Andrew C. Slagter, Drew VanWoerkom, Derby Effie Addison, Samantha M. Arteaga, Rory E. Barnard, Nick M. Bultje, Junu Chung, Brenna M. Coleman, Cameron D. Figg, Audrey J. Gonzales, Josiah M. Hoekman, Kat H. Irmen, Valerie G. Krause, Natalie L. Luginbill, Rebecca A. Nulty, Alexander W. Scofield, Marguerite C. Sytsema, Charles E. Tavera, Anna G. Thomas, Simon P. Troyer, Hannah Sheppard, Myles D. Radersma, John T. Wertz, Randall J. DeJong

**Affiliations:** 1 Calvin University, Grand Rapids, Michigan, United States

## Abstract

We report genomes of five phages isolated using the actinobacterium *Rhodococcus equi* NRRL B-16538 *.* Based on gene content similarity, one phage is assigned to actinobacteriphage cluster CF, one to cluster CR, two to cluster CG, and one that cannot be assigned to any existing cluster. The latter encodes a five-gene thymine hypermodification system.

**Table 1. Characteristics of five phages isolated from Rhodococcus equi NRRL B-16538:** ^a^ Minimum 3 phage particles measured

**Table:** 

Phage Name	CoffeeBean	Dorin	Francesca	Madraxi	Tonitrus
Soil sample collection site	Grand Rapids, MI, 42.931694 N, 85.581889 W	Grand Rapids, MI, 42.928267 N, 85.587137 W	Grand Rapids, MI, 42.93455 N, 85.58755 W	Grandville, MI, 42.897778 N, 85.737222 W	Grand Rapids, MI, 42.93155 N, 85.586913 W
Capsid size (nm)<sup>a</sup>	62-64	75-80	69-88	58-60	56-81
Tail length (nm)<sup>a</sup>	243-250	333-355	353-439	238-242	268-323
No. of 150 bp sequencing reads	2,000,000	2,000,000	2,000,000	856,305	482,745
Average shotgun coverage (fold)	194	104	296	1862	1081
Genome length (bp)	67362	136968	137748	64064	57971
Genome ends	3' single-stranded overhang,<br/>5' AGCCGCGTAC	direct terminal repeat (4720 bp)	direct terminal repeat (4862 bp)	3' single-stranded overhang,<br/>5' CCCGCC	circularly permuted
G + C content (%)	67.3	48.0	48.0	66.2	69.0
No. of ORFs	95	282	281	108	84
Cluster	CR	CG	CG	CF	Singleton
No. of tRNA-coding regions	0	33	33	0	0

## Description

*Rhodococcus equi* is an Actinobacteria commonly found in soil and known to cause lung infections in livestock and immunocompromised humans (Weinstock & Brown, 2002). Limited phages capable of infecting *Rhodococcus equi* have been documented ( Summer et al., 2010; Bonilla et al., 2017; Radersma et al., 2024 ) . Here, we report on five phages isolated in 2023 using *R. equi* NRRL B-16538 and standard methods (Zorawik et al., 2024). Soil samples collected from ~ 5 cm below the soil surface were washed in PYCa broth and filtered (0.22µm pore size). Directly plating the filtrate in PYCa top agar with *R. equi* yielded phages Coffeebean and Madraxi. Incubation of the filtrate with *R. equi* for 2 days at 30˚C before refiltration and plating yielded Dorin, Francesca, and Tonitrus. All phages have siphovirus morphologies, as determined by transmission electron microscopy.

Phage DNA was extracted from lysate using a Qiagen DNeasy kit, then prepared for sequencing using the NEBNext Ultra II-FS DNA library prep kit. Sequencing was performed with an Illumina MiSeq (v3 reagents), yielding 150-base single-end reads that were assembled using Newbler v2.9 and Consed v29 with 104 to 1862-fold shotgun coverage (Miller et al., 2010; Gordon & Green, 2013; Russell, 2018). Genomes were then annotated using DNA Master v5.23.6 (Pope & Jacobs-Sera, 2018), PECAAN v20221109 (Rinehart et al., 2016), Glimmer v3.02 (Kelley et al., 2012), Genemark v2.5 (Besemer et al., 2001), Phamerator (Actino_draft database v578) (Cresawn et al., 2011), BLASTp v.2.14.1 (Actinobacteriophage and NCBI non-redundant protein databases) (McGinnis & Madden, 2004), HHPred (PDB, UniProt, Pfam-A v.36, and NCBI v.3.19 databases) (Söding et al., 2005), Aragorn (Laslett & Canback, 2004), tRNAscanSE v2.0 (Lowe & Eddy, 1997), DeepTMHMM v1.0.24 (Hallgren et al., 2022), and TOPCONS v2.0 (Tsirigos et al., 2015), all using default parameters. Sequencing details and genomic characteristics are provided in Table 1.

Phages were assigned to clusters based on gene content similarity (GCS) of at least 35 % (Pope et al., 2017) to phages in the actinobacteriophage database (https://phagesdb.org/). Phage Tonitrus did not meet this threshold and is therefore classified as a singleton. The first five genes of Tonitrus may be associated with a thymidine hypermodification (THM) system, including a parB-like nuclease domain, a tet-like J-binding protein, an aGPT-Pplase2 domain protein, a 5-hmU DNA kinase, and a hypothetical protein (Lee et al., 2018, 2022). This system may defend against bacterial restriction endonucleases through chemical modification of thymidine (Lee et al., 2018, 2022; Flodman et al., 2019). Phage CoffeeBean is assigned to cluster CR, which is principally composed of phages isolated using *Gordonia* ; to date, only seven of forty-nine cluster CR phages were isolated using *R.equi* (Radersma et al., 2024). Phages Dorin and Francesca share 91% GCS, are assigned to cluster CG, and contain 33 tRNA-coding regions each. Phage Madraxi is assigned to cluster CF and is the only phage presumed to be a temperate phage based on the presence of identifiable serine integrase and immunity repressor functions; these functions could not be identified in the other four phages. Except for Tonitrus, a translational frameshift in the tail assembly chaperone protein was identified for the other phages reported here.

**Data availability.** Annotated genome sequences can be accessed for CoffeeBean, Dorin, Francesca, Madraxi, and Tonitrus at GenBank accession numbers PP978888 , PQ114736 , PP978770 , PP978820 , PP978825 , respectively. Sequence reads are deposited at NCBI under SRA accession numbers SRX25029070 , SRX25029071 , SRX25029053 , SRX25029059 , SRX25029066 .
